# Fast time-domain diffuse correlation spectroscopy with superconducting nanowire single-photon detector: system validation and in vivo results

**DOI:** 10.1038/s41598-023-39281-5

**Published:** 2023-07-24

**Authors:** Veronika Parfentyeva, Lorenzo Colombo, Pranav Lanka, Marco Pagliazzi, Annalisa Brodu, Niels Noordzij, Mirco Kolarczik, Alberto Dalla Mora, Rebecca Re, Davide Contini, Alessandro Torricelli, Turgut Durduran, Antonio Pifferi

**Affiliations:** 1 Institut de Ciéncies Fotòniques, The Barcelona Institute of Science and Technology, Castelldefels, Barcelona, 08860 Spain; 2 Dipartimento di Fisica, Politecnico di Milano, Milan, 20133 Italy; 3 Single Quantum BV, Delft, 2629 JD The Netherlands; 4 Swabian Instruments GmbH, Stuttgart, 70435 Germany; 5 Consiglio Nazionale delle Ricerche, Istituto di Fotonica e Nanotecnologie, Milan, 20133 Italy; 6 Institució Catalana de Recerca i Estudis Avançats (ICREA), Barcelona, 08015 Spain

**Keywords:** Optics and photonics, Physics

## Abstract

Time-domain diffuse correlation spectroscopy (TD-DCS) has been introduced as an advancement of the “classical” continuous wave DCS (CW-DCS) allowing one to not only to measure depth-resolved blood flow index (BFI) but also to extract optical properties of the measured medium without using any additional diffuse optics technique. However, this method is a photon-starved technique, specially when considering only the late photons that are of primary interest which has limited its in vivo application. In this work, we present a TD-DCS system based on a superconducting nanowire single-photon detector (SNSPD) with a high quantum efficiency, a narrow timing response, and a negligibly low dark count noise. We compared it to the typically used single-photon avalanche diode (SPAD) detector. In addition, this system allowed us to conduct fast in vivo measurements and obtain gated pulsatile BFI on the adult human forehead.

## Introduction

Diffuse correlation spectroscopy (DCS) is an optical technique for the non-invasive monitoring of the deep tissue ($\sim \,1$ cm) micro-vascular blood flow in biological tissues^1^. The technique measures the temporal fluctuation of coherent light multiply-scattered by the sample due to the diffuse speckle fluctuations. The intensity temporal auto-correlation function is typically used to quantify this fluctuation—its decay rate being related to the motion of the scattering centers^2,3^. In the case of biological tissues, the moving scatterers are mainly red blood cells. Thus, from the decay rate of the intensity auto-correlation function, it is possible to extract a blood flow index (BFI), which has been shown to be highly correlated with micro-vascular blood flow^1,4–6^.

Since DCS generally uses a continuous-wave laser, photons with any possible path lengths contribute to the measurement without any discrimination. For this reason, unless multiple source-detector separations are used, the technique typically lacks depth resolution^7^. Another limitation of DCS is that a separate measurement of the optical properties (absorption and reduced scattering coefficient) is necessary for an accurate BFI estimation^8^. Other techniques, such as time-domain near infrared spectroscopy (TD-NIRS), by exploiting the physical relationship between the photon path length (or equivalently the time-of-flight) and the average penetration depth, obtain depth-resolved measurements for the optical properties, but lack information on blood flow^9^. A hybrid TD-NIRS and DCS system can be used to retrieve both optical and dynamic properties, but at the cost of increased complexity and bulkiness.

This principle is exploited by time-domain DCS (TD-DCS), a novel technique which uses pulsed laser sources^10–14^ and measures the path length (or time)-resolved speckle intensity auto-correlation functions. The time-resolved acquisition strategy might enable obtaining a depth-resolved blood flow information, as well as the parallel estimation of the optical properties, which are extracted from the measured distribution of time-of-flights (DTOF) curve, thus without the need of additional measurements. The two main challenges of the technique are: (1) the need of a pulsed laser (with tens/hundreds ps duration) with high temporal coherence^15,16^; and (2) the constraint of single-mode detection, which limits light harvesting.

TD-DCS was demonstrated in 2016 by Sutin et al.^12^, with phantoms and in vivo experiments on rodents. Shortly after, Pagliazzi et al., reported the first in vivo experiments with human volunteers^13,17^ and later on a portable instrument was also developed for human studies^18,19^. Recently, researchers have also demonstrated the use of longer wavelengths, in particular beyond 1 $\upmu$m, for increasing the signal-to-noise ratio of the measurements^20^. In parallel, several theoretical studies have been reported, with the aim of optimizing the acquisition strategy^21,22^, or improving the data analysis^16,23^. Despite these efforts, the technique still suffers from limited depth sensitivity, which is crucial for instance in measurements of the cerebral hemodynamics, limiting its translation to the clinics.

In this work, to optimize the detection performance, we report a TD-DCS system based on a superconducting nanowire single-photon detectors (SNSPD)^24^. SNSPDs are a relatively new class of photo-detectors, which exploit current-biased superconductive filaments to detect photons with an enhanced efficiency compared to semiconductor-based photo-detectors (up to $\sim \,99\%$^25^). Further, they exhibit negligibly low dark count noise (few counts per second -cps-), fast afterpulsing decay time constant ($\sim$ ns)^26^, small temporal jitter ($<5$ ps^27^), and absence of slow decaying tails in their single-photon timing response. For these reasons, SNSPD have been used extensively in several research fields such as quantum optics and optical telecommunications^28^. Few groups, including our own, have recently explored the usability of SNSPD for DCS and in particular TD-DCS^19,29–32^. Each realisation presented different embodiment and laser source. In this work we will present the system developed in the joint POLIMI-ICFO lab based on a unique laser source suitable for both adjustment of pulse-width/temporal coherence and wavelength tunability from 700 to above 1000 nm.

## Materials and methods

### Experimental setup

**Figure 1:**
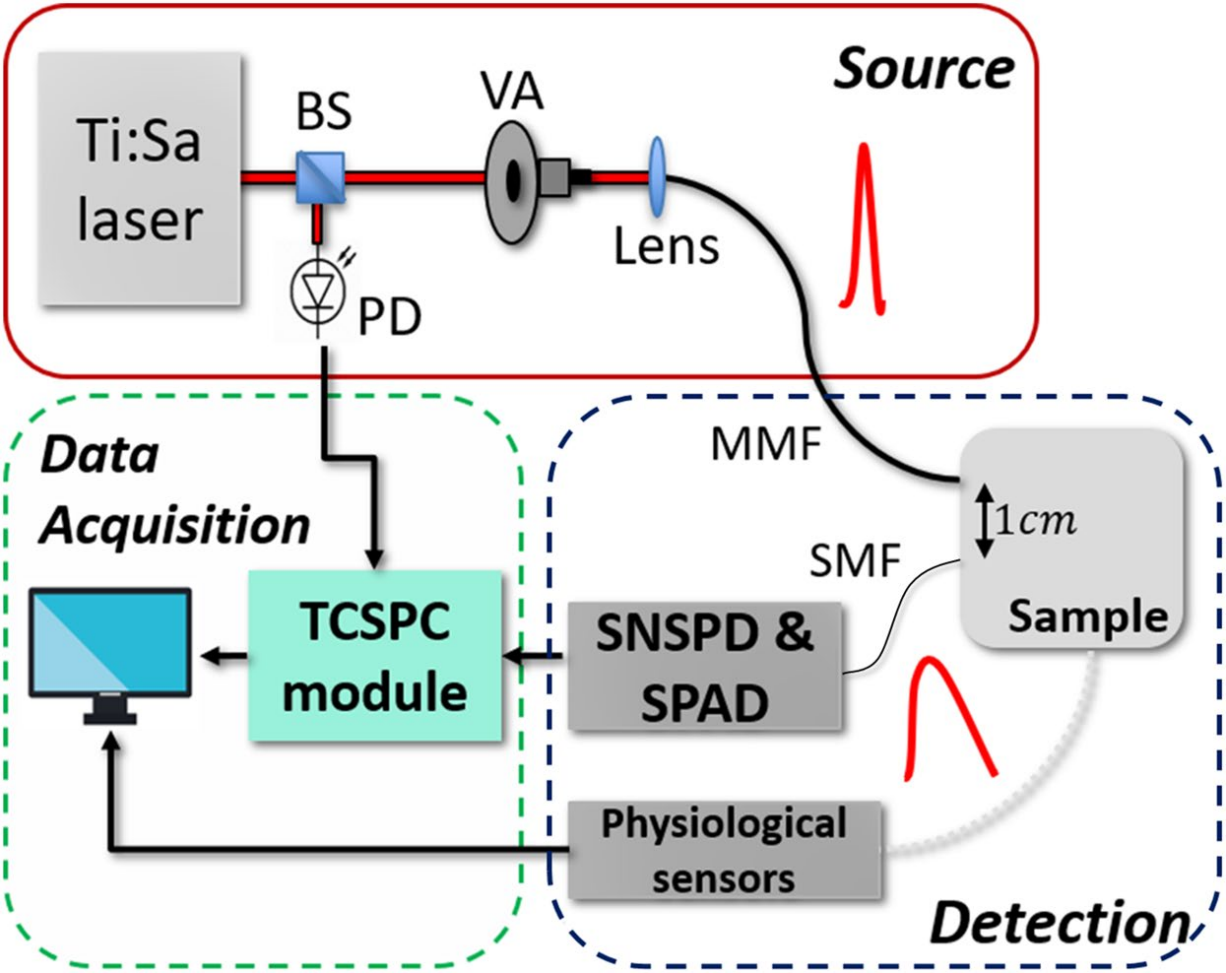
Experimental system (BS, beam splitter; VA, variable attenuator; PD, photodiode; MMF, multimode fiber; SMF, single mode fiber; TCSPC, time-correlated single-photon counting), schematically divided in source, detection and data acquisition units (red, grey and green boxes respectively).

The experimental setup is shown schematically in Fig. 1. It is logically divided in source, detection and data acquisition units (red, blue, and green blocks, respectively).

The source unit is composed of a custom mode-locked Ti:Sapphire laser with variable wavelength and pulse width^13^. In this work we adopted a wavelength ($\lambda$) of 785 nm, a pulse duration of $\sim \,\,100-200$ ps and 100 MHz repetition rate. By means of a beam splitter (BS), a small part of the laser power ($< 5 \%$) is delivered to a photodiode (PD) (OCF-401, Becker & Hickl, Germany) for generating a synchronization signal. The remaining part, after passing through a variable attenuator (VA), is coupled to a graded index, 100 $\upmu$m core diameter, multi-mode fiber (MMF) and delivered to the sample.

In the detection unit, the diffusely reflected photons are collected by using a single-mode fiber (SMF, Thorlabs, Germany) at a source-detection separation of $\rho = 1$ cm. The detection fiber is connected to an SNSPD detector (Single Quantum BV, The Netherlands) optimized for $\lambda \sim \, 800$ nm. At $\lambda \sim \,780$ nm, this detector exhibits a photon detection efficiency of $\sim \,70\%$ for unpolarized light (resulting from an efficiency of $85\%$ for polarized light). Additionally, its dark count rate is 10 cps, afterpulsing is absent, timing jitter is 24 ps, and exponential decay tails in the detector single-photon response are absent. Its output pulse amplitude is 870 mV and the electric dead time is 9.9 ns. For maintaining the SNSPD in the superconducting state, it is placed in a cryostat which keeps a temperature $T< 3$ K by means of a dedicated closed-loop helium compressor (CNA-11, Sumitomo Heavy Industries Ltd, Japan). Another identical detection fiber, collecting light at the same source-detector separation, is coupled with a SPAD (PDM, Micro Photon Devices, Italy). At $\lambda \sim \,780$ nm, this detector exhibits a photon detection efficiency of $\sim \,15\%$. Additionally, its dark count rate is $< \,100$ cps, afterpulsing is $< \,3\%$, timing jitter is $< 50$ ps, and the exponential decay tail in the detector single-photon response has a $> \,200$ ps time constant, thus enlarging its response shape with respect to the pure gaussian jitter contribution. Its output pulse amplitude is 800 mV and the electric dead time is 77 ns. For the in vivo experiments, additional physiological sensors were connected to the system, namely: 3-leads electrocardiogram (ECG) and respiration chest band (FlexComp, Thought Technology Ltd, Canada). The physiological sensors were synchronized with the TD-DCS system by means of a periodic (1 Hz) TTL reference clock signal generated by the computer (PC).

In the data acquisition unit, the signals from the detectors, together with the synchronization signal, are acquired with a time-correlated single-photon counting (TCSPC) module (Time Tagger Ultra, Swabian Instruments GmbH, Germany). For every detected photon, the absolute arrival time and time-of-flight (10 ps resolution), combined with the channel number, are stored continuously on the PC.

### Phantom experiments

For characterization of the system and comparison of SNSPD to SPAD, we prepared a tissue-mimicking phantom by adding $5\%$ in mass of Intralipid 20 (B. Braun Melsungen, Germany) to distilled water. The nominal optical properties were $\mu '_s = 10$ $\textrm{cm}^{-1}$ and $\mu _a = 0.1$ $\textrm{cm}^{-1}$ at $\lambda = 785$ nm^33^. The phantom was measured in parallel with the SNSPD and the SPAD detectors, therefore possible differences in the measured signals are due to the difference in the performance between detectors.

### In vivo experiments

We have performed in vivo experiments on adult volunteers with the protocol approved by the ethical committee of Politecnico di Milano (“parere n 37/2020”) and have recruited four volunteers (see Table 1). The research has been performed in accordance with relevant regulations and with the Declaration of Helsinki. The informed consent was obtained from all volunteers. The probe was placed on the forehead of the subject at the FP1 position according to the 10/20 EEG international system^34^. In addition, the physiological sensors recording the electrocardiogram (ECG) and the chest (thoracic) expansion/contraction at 256 Hz were used. For ECG, we placed one electrode under right (one under the left) clavicle, mid-clavicular line within the rib cage frame. The third electrode was placed on the left lower abdomen. We have asked the subject to lay on a bed with backrest tilted at 45 degrees. All measurements were done only using the SNSPD detection channel.

**Table 1:** Demographic data of the subjects and fitted optical properties for all the subjects.

	S1	S2	S3	S4
Age	52	37	56	51
Sex	M	F	M	M
$\mu '_s [\textrm{cm}^{-1}]$	12.3	12.1	13.1	12.2
$\mu _a [\textrm{cm}^{-1}]$	0.08	0.10	0.19	0.14

First, we have performed a *Resting state* protocol (*Protocol 1*), where the subject was asked to stay supine, breathing at their normal breathing rate and depth, with their eye closed for seven minutes.

In the second part (*Protocol 2*), we have asked the participants to perform a *Valsalva maneuver* (VM)^35–37^ by blowing into an empty straw, which was closed at the far end. We chose this exercise because previously it was used to separate intra- versus extra-cerebral signals^37^ meaning that, potentially, VM induces changes in hemodynamics which are different for later time gates compared to the earlier ones. The protocol was as follows: (1) baseline (80 *s*), (2) Valsalva maneuver (20 s), (3) normal breathing (160 s). Before VM, subjects were asked to do few trial repetitions before the start of the protocol to make sure they perform properly. In total, each subject performed three VM exercises.

**Figure 2:**
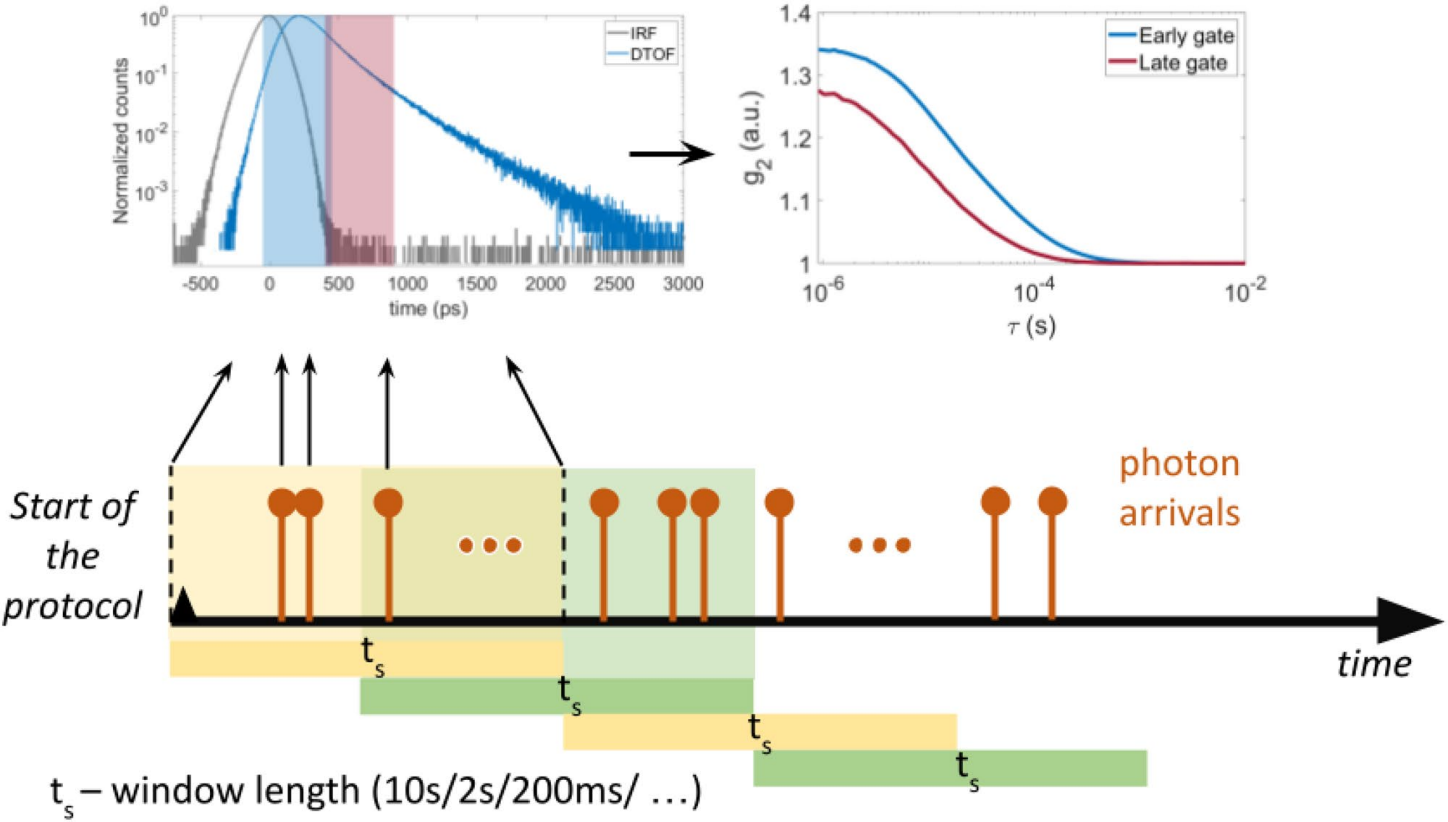
Scheme on how photon arrival times are registered, windowed with overlapping segments and then processed with calculating intensity autocorrelation function.

### Instrument response function acquisition

Each measurement described above, either phantom or in vivo, was followed by acquiring instrument response function (IRF). This was done by facing the tips of source and detection fibers with a static diffuser in between. Each IRF measurement was 10 s long.

### Data analysis

The overall file of detected photons, often called time-stamps, is then post-processed with a Python-based software (SW) correlator^13,17,23^ to obtain DTOF and IRF curves and (time-gated) auto-correlation functions (ACF) with a flexible sampling time (typically between 0.1 and 10 s). For the computation of the auto-correlation curves, we have adapted the algorithm proposed by Wahl et al.^38^ for frequency correlation spectroscopy.

From the measured intensity ACF $g_2(\tau )$, the electric-field ACF $g_1(\tau )$ is modeled by means of the so-called Siegert relation^39,40^:

1 $$\begin{aligned} g_{2}(\tau ) = 1 + \beta \left| g_{1}(\tau )\right| ^2, \end{aligned}$$

where $\beta$ is a system-dependant factor, so-called coherence parameter, which is inversely proportional to the number of collected modes. Its maximum value is 1/2 for unpolarized light.

In Eq. (1), the electric-field ACF might be modeled as^16,23^:

2 $$\begin{aligned} g_{1}(\tau ) = \int _{0}^{+\infty } EGF(s)P(s) exp \left( -2 \mu _{s}^{\prime } k_0^2 \alpha D_{B} s \tau \right) ds \end{aligned}$$

where $\mu _{s}^{\prime }$ is the reduced scattering coefficient of the sample, $k_0$ is the light wave-number in the medium, $\alpha$ is the fraction of moving to total scatterers, and $D_B$ is their Brownian diffusion coefficient. In case of in vivo studies, $\alpha D_B$ is reported as a blood flow index (BFI) and was shown to be an accurate measure of microvascular blood flow as discussed above. Also, $P(s=vt)$ is the (ideal) path length probability distribution (*v* being the speed of light in the medium), estimated with the theoretical time-resolved diffuse reflectance^41^. Finally, in Eq. (2), *EGF*(*s*) is the so-called effective gate function, defined as:

3 $$\begin{aligned} EGF(s) = \int _{s_0}^{s_0+\Delta s} IRF(s'- s) d{s'}, \end{aligned}$$

where $IRF(s=vt)$ denotes the instrument response function (with its peak position defined as $t_0=0$), $s_0=t_0 v$, and $\Delta s=\Delta t v$ represent the gate start and gate width (respectively).

For the data analysis, the measured $g_2(\tau )$ is fitted with respect to the $\alpha D_{B}$, which is defined as blood flow index (BFI). The fitting is performed in MATLAB by minimizing the squared error between the measured and theoretical $g_2(\tau )$ (Eq. 1 coupled with Eq. 2). The fitting region was from $\tau = 10^{-6}s$ to the point were $g_1(\tau )$ falls below 0.5. Optical properties retrieval was done using model described here^42^.

In the analysis of the phantom measurements, for basic instrument performance assessment, the measured IRF curves were normalized by their maximum and, together with the corresponding DTOF curves, were shifted so that the IRF peak was located at a zero time. Integration time for both DTOF and IRF was 10 s. For performance assessment in TD-DCS, photon time stamps were processed with the SW correlator with a sampling rate of 0.1 Hz for a total acquisition time of 300 s (therefore, giving 30 autocorrelation curves). Additionally, for SNSPD measurements, we fitted $\alpha D_{B}$ for 200 ps wide time gates positioned at different parts of the DTOF using model described above in order to evaluate our fitting algorithm.

Concerning in vivo data of *Protocol 1*, optical properties have been retrieved for each subject. For this purpose we have fitted an experimental DTOF obtained by averaging a 10 s chunk of the seven minutes of measurement. The experimental IRF has been acquired after the in vivo measurement with a 10 s integration time. In the post processing stage with the SW correlator, the recorded photon time stamps were sampled with 50% overlapping windows ($t_s$) of 200 ms length and $g_2(\tau )$ were calculated for early and late gates, as schematically represented in Fig. 2. Sampling with overlapping windows helped to achieve higher amount of photons per time window without significantly sacrificing the time resolution which has to be high enough to resolve BFI pulsatility. Note that in this work overlapping windows are applied before gating of photons according to their time of arrival, therefore, it does not affect results of comparison between early and late gate signals. For each subject, the early and late time gates were chosen to be 500 ps wide gates starting at $-\,50$ ps and 400 ps with respect to the IRF. These gates were chosen as a trade off between longer time-of-flight and minimal signal-to-noise ratio (SNR) in the late gate allowing to resolve pulsatile BFI. Then, BFI was derived by fitting $g_2(\tau )$ curves with the model described previously. For further analysis BFI was normalized by the mean BFI value of the entire measurement to get relative BFI (rBFI). We have performed a frequency analysis of the rBFI signal using MATLAB’s *pwelch* function based on the Welch algorithm, with Hamming windows of 2 minutes length without overlap.

In vivo data of *Protocol 2* were analysed by using the same time gate positioning as for *Protocol 1* experiment. The sampling was done with time windows ($t_s$) of 2 s length and an overlap of 50% (slow) and with 200 ms length and 50% overlap (fast). First, each VM repetition was evaluated by looking at the BFI time trace and at the physiological sensor’s data (particularly at the respiratory and heart rates derived from chest belt and ECG recordings respectively by calculating inverse of peak-to-peak distance with sliding window averaging) and got accepted or rejected for the further analysis. For each chosen VM (20 s of the exercise ± 20 s margins), relative BFI was calculated by dividing BFI by the mean of the 1 minute period prior to the particular VM repetition. Then, these rBFI time traces were aligned and averaged to compare the group response at different time gates. Additionally, we defined 4 VM phases similarly to how it was done in^35,36^ and compared hemodynamic response between early and late gates separately for each phase.

## Results

### Phantom measurements

**Figure 3:**
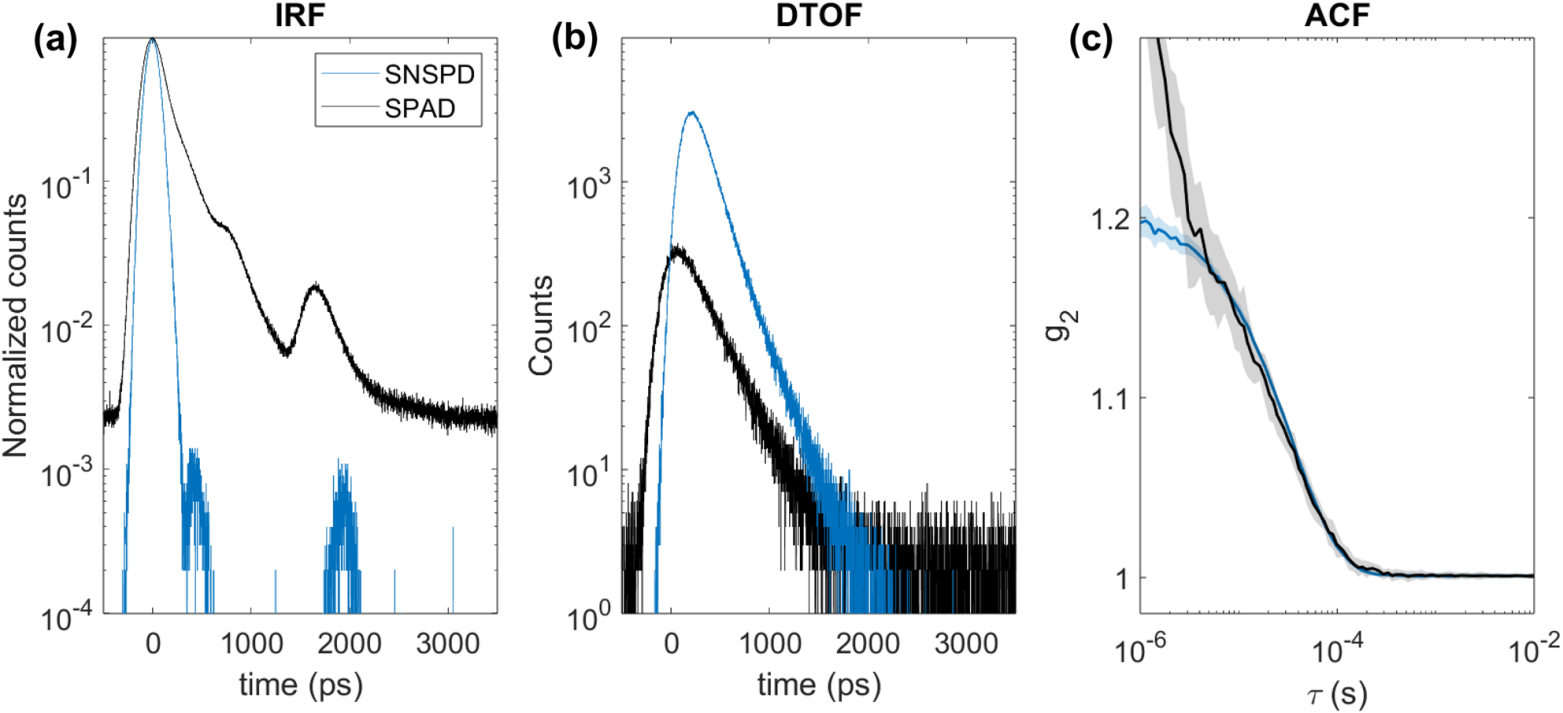
Intralipid phantom measurement with the SPAD detector and the SNSPD: black—results from the SPAD, blue—results from the SNSPD; (**a**) measured normalized IRF, (**b**) measured DTOF, (**c**) ungated auto-correlation function averaged over 30 curves, shaded region represents a standard deviation.

The measured IRF, DTOF and calculated ungated ACF are represented in the Fig. 3. Both IRF and DTOF (Fig. 3a, b) of the SNSPD demonstrate almost no background while the IRF of the SPAD detector is comparably wider, in particular because of the evident exponential tail in the response that affects late times. Both IRFs exhibit secondary peaks due to reflections between the laser output and optical elements in the free beam. Moreover, in Fig. 3c, $g_2$ curves of the SPAD detector at very low correlation times ($10^{-6}$ s) clearly manifest an artificial increase of the $g_2$, which is expected due to afterpulsing noise. This phenomenon is totally absent for SNSPD. Table 2 summarizes the characteristics of the measured signals for both the SPAD detector and the SNSPD.

**Table 2:** Comparison of the signal characteristics between SPAD and SNSPD.

Detector	IRF FWHM (ps)	IRF FW at 10% (ps)	IRF FW at 1% (ps)	Count rate (kcps)
SPAD	250	650	2140	20
SNSPD	160	300	440	120

**Figure 4:**
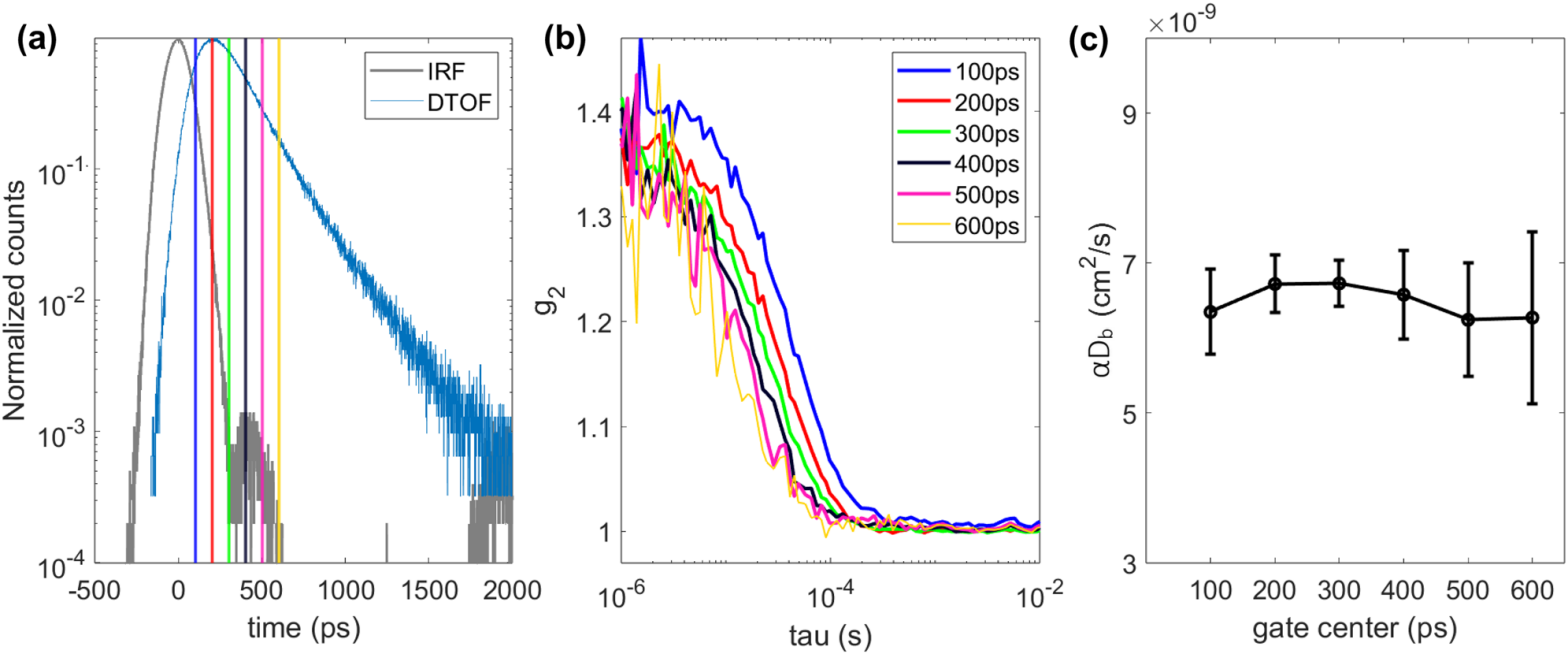
Gated analysis of the intralipid phantom measurements with the SNSPD. (**a**) IRF (gray) and DTOF (blue) of the SNSPD with gates, (**b**) example of one $g_2$ curve for each gate, (**c**) fitted $\alpha D_{b}$ averaged over 30 values with standard deviation for each gate.

Additionally, in case of SNSPD measurements, we have looked at the $\alpha D_{B}$ (in a liquid phantom $\alpha \sim 1$) for the time gates positioned at the different parts of the DTOF. Figure 4a shows the positioning of the 200 ps wide gates. Single auto-correlation curves and fitted $\alpha D_{B}$ for each gate are shown in Fig. 4b, c. $g_2$ curves have different decay rates and $\beta$ due to the different position of the gate (probed depth) which has been observed before in^13,23^, however, $\alpha D_{B}$ derived from these curves is the same for all the gates which is due to the fact that we were measuring a homogeneous phantom. This confirms that our fitting algorithm is not affected by the gate positioning, and that we properly account for the IRF.

### In vivo experiments

#### Resting state

**Figure 5:**
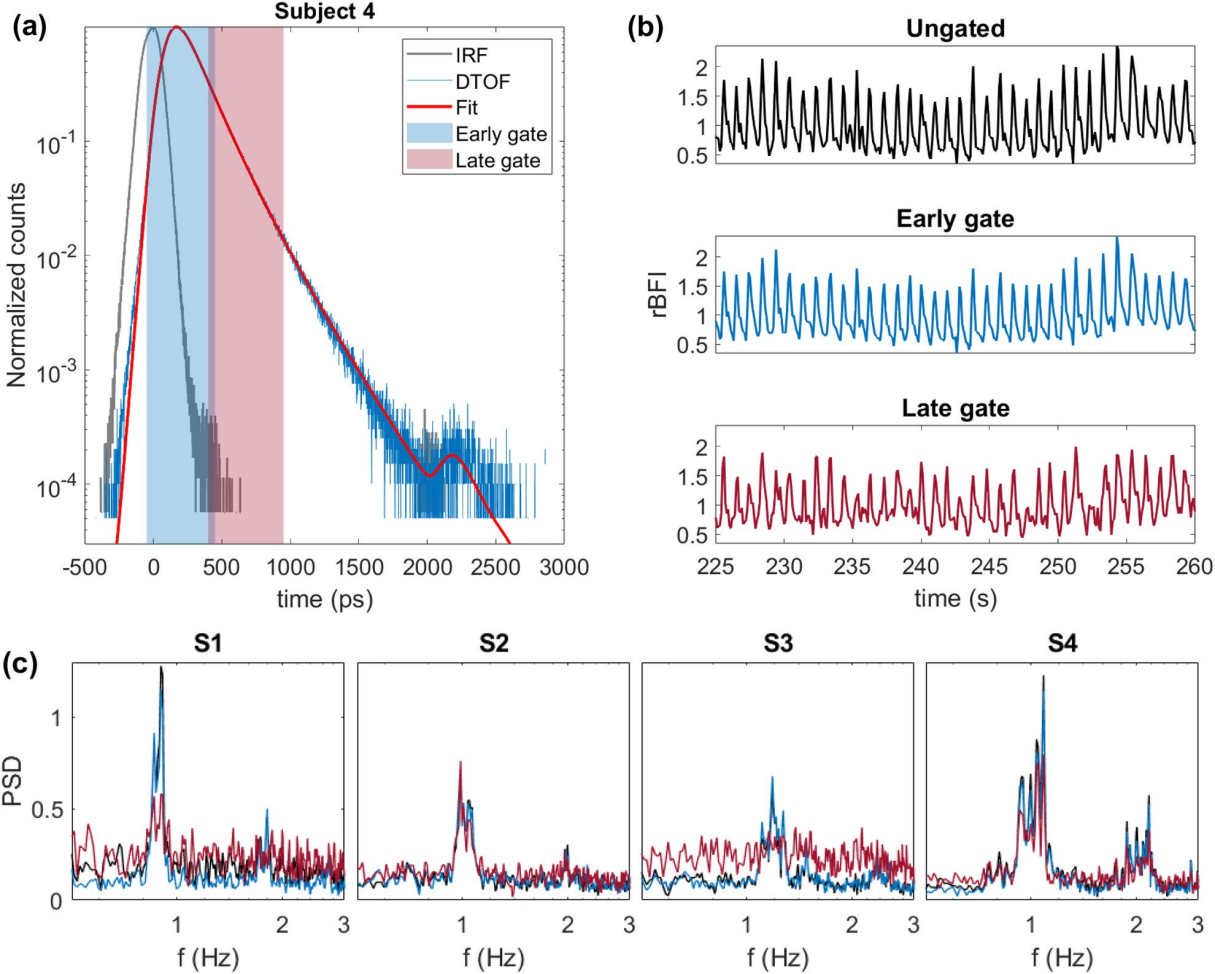
(**a**) IRF (gray), DTOF (blue), and fitted DTOF curve (red) of Subject 4 with shaded early and late gates; (**b**) pulsatile rBFI of Subject 4; (**c**) Power spectral density estimate (PSD) calculated over 7-minute of *Resting state* period for all 4 subjects. Colours correspond to different gates: black—ungated case, blue—early gate, red—late gate.

The optical properties derived for each subject are summarized in Table 1. Figure 5a, b show the gate positioning and example of rBFI for different gates for Subject 4. We demonstrate a fitted DTOF curve obtained as a convolution of the experimental IRF and the theoretical reflectance calculated using fitted optical properties. As can be seen from Fig. 5b, Subject 4 demonstrates a good SNR which allows one to resolve pulsatility even for a late gate case, which typically suffers from lack of photon counts. Similar plots for the rest of the subjects are shown in the Supplementary Materials (Fig. S1). The obtained frequency spectrum for each subject is shown in Fig. 5c. All the subjects have a heart rate peak around 1 Hz in ungated and early gate signals, however, Subject 3 does not demonstrate the heart rate peak in the late gate signal due to the low photon counts for this gate. Count rate for all the subjects and time gates are shown in the Table S1 in the Supplementary Materials.

#### Valsalva maneuver

**Figure 6:**
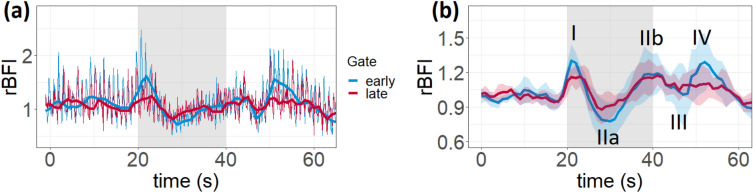
(**a**) Example of slow (bold line) and fast (thin pulsating line) rBFI signals to VM exercise (grey region highlights the VM time period); (**b**) rBFI response to VM exercise averaged over 6 repetitions (2 subjects $\times$ 3 repetitions), numbers show the location of each phase.

As it was mentioned previously, each subject performed three repetitions of the VM exercise. Based on the results from the physiological monitoring and the quality of TD-DCS signal, we chose two subjects which demonstrated a clear response to the exercise during all three repetitions. For each repetition of VM, rBFI was calculated, as described in the “Materials and methods” section. Figure 6a shows examples of rBFI response for “fast” (200 ms windows with 50% overlap) and “slow” (2 s windows with 50% overlap) sampling rates. We averaged slow rBFI responses from six repetitions and results are shown in Fig. 6b. For each phase shown in Fig. 6b we calculated rBFI values for early and late gates. Additionally, we looked at the changes of pulsatility parameters, pulsatility index (PI) and pulse height (PH), derived from BFI during VM. After the start of VM, PI drops by 52 ± 7% and 31 ± 9% at early and late time gates respectively and PH drops by 55 ± 10% and 41 ± 5% respectively.

## Discussion and conclusions

We have demonstrated fast in vivo TD-DCS acquisitions thanks to the use of a high-sensitive and narrow-response SNSPD-based detection system. We have tested the SNSPD-based TD-DCS system and compared it to the “classical” SPAD detector in the context of phantom experiments. In this part, data have been processed with SW correlator with the sampling rate of 0.1 Hz. Results demonstrate that SNSPD has a narrower IRF, six times higher count rate and negligible background noise comparing to the SPAD detector. Moreover, ACFs measured with SNSPD have a smaller standard deviation and do not reveal any afterpulsing effect which is clear form Fig. 3. The summary of SNSPD and SPAD signal characteristics is presented in Table 2.

On the data from the same phantom experiment we have conducted gated analysis in order to validate our fitting model. In the future, direct gated acquisition acting on the SNSPD may also become possible thanks to recent developments^43^. However, the SNSPD gating speed is still insufficient for this applications and, additionally, it could result beneficial only when the ungated detector is saturated at full laser power, which is not the case for the present work. Data recorded with SNSPD have been gated with 200 ps wide gates centered at positions of the DTOF (see Fig. 4a) and ACF and $\alpha D_{B}$ have been calculated for each time gate using the model described in^23^. We have observed that, with increasing gate time, $\beta$ gradually decreases and ACF gets noisier which is in accordance with the previous findings^12,13^. Conversely, $\alpha D_{B}$ does not significantly change with the time gate, which follows our expectations for a homogeneous liquid phantom.

The system as a whole, and in particular the detector, can have a major impact on: (1) SNR of the autocorrelation curve $g_2$; (2) probed depth; (3) spatial resolution. It is commonly accepted that the SNR of the $g_2$ scales linearly with the number of detected photons per independent speckle, and with the square root of the number of detectors. Thus the higher quantum efficiency—almost a factor of 5 in our case—directly translates in significant improvement in the $g_2$, as observed in Fig. 3c. In turn, the higher quantum efficiency permits to reach a larger maximum photon time-of-flight and therefore a larger depth. In addition, depth sensitivity is also increased due to the lack of early (i.e. shallow) photon contamination, which is present instead in SPADs^44^. Finally, spatial resolution can be improved by operating at null or short source-detector distances. While in principle this condition leads to higher signal at any photon arrival time, higher sensitivity, and better spatial resolution^45^, in practice the benefits must be weighted against the stronger impact of the IRF on the detected signal due the the overwhelming burst of early photons. The almost ideal IRF of the SNSPDs and the high count rate tolerated by the detector permits to disentangle the influence of early photons and naturally enable short distance measurements. A systematic quantitative comparison between SPADs and SNSPDs for TD-DCS on application-specific figures-of-merits is too premature at this stage. It will be possibly pursued in a following activity with growing experience in TD-DCS and formulation of shared metrics for performance assessments.

For in vivo experiments, high SNR and DTOF resolution of our experimental setup with SNSPD allowed us to use all capabilities of the TD-DCS technique and not only extract gated BFI but also derive optical properties for each subject individually which then were used in the fitting algorithm unlike similar works^19,46^ on TD-DCS-SNSPD systems using fixed optical properties for all subjects. It is worth mentioning that all in vivo measurements have been analysed using same early and late gates temporal positioning for all subjects, thus not considering possible variability between them. In other words, while for one subject late gate starting at 400 ps could probe deeper brain region, in another one it might not. One possible improvement (intrinsically offered by the time-domain approach since the arrival time of detected photons encodes the average investigated depth^9^) could be the use of DTOF derived optical properties and/or magnetic resonance imaging derived information on the head geometry, which could both lead to defining optimal gate parameters on an individual basis.

For *Protocol 1*, we have processed photon time tags with SW correlator windowing them with 200 ms overlapping windows (50% overlap) in order to achieve equivalent to 10 Hz sampling rate and resolve pulsatile BFI. For all subjects we used the same gate configuration shown in Fig. 5a. The late gate has a width of 500 ps and starts at 400 *ps* with respect to the peak of IRF (or about 300 ps from the peak of the DTOF). This late gate configuration is close to the optimal one at 765 *nm* reported by^22^ for TD-DCS monitoring of cerebral blood flow, where they claim that gate start times varying from 300 to 400 ps with respect to the peak of temporal point spread function and widths of 500 ps and above correspond to both high contrast-to-noise ratio and high intrinsic sensitivity. We report that for three out of four subjects the frequency spectrum of “fast” rBFI has a clear heart rate peak around 1 Hz for both early and late time gates which matches HR derived from the ECG data. Subject 3 has, in general, a lower count rate comparing to the rest on the subjects and does not show a clear heart rate peak in the frequency analysis for the late time gate. Both conditions are consistent with the high scattering and absorption coefficients leading to a low number of photon counts at the late gate. In the scope of this work, we demonstrated PSD spectrum calculated over the duration of the baseline protocol which only represents an average feature of the pulsatile BFI, but it would be interesting to look at the dynamic changes in the rBFI waveforms over time for different time gates during various hemodynamic stimulus. This would require even higher signal SNR to be able to extract gated rBFI at higher sampling rates.

For *Protocol 2*, we looked at the rBFI of different time gates sampled with 2 s windows (slow) and 50% of overlap and with 200 *ms* windows and 50% of overlap (fast). An example of rBFI response processed with fast and slow sampling rate is shown in Fig. 6a and the averaged rBFI response over 6 VM repetitions from 2 subjects is shown in Fig. 6b. The observed hemodynamic response to VM is in accordance with the transcranial doppler ultrasound studies which used *Valsalva maneuver* as a cerebral hemodynamics stimulus^36,37^. For analysis, rBFI response to VM was divided into 4 phases shown in Fig. 6b similarly to the studies mentioned above. During phase I, the subjects begin forcefully exhalating against a closed airway, the intrathoracic pressure suddenly increases which leads to the brief increase in the systemic arterial blood pressure (ABP) and cerebral bloof flow. In phase IIa^47^, the systemic ABP, first, decreases (IIa) since the cardiac filling is reduced and then rises from its nadir. At the immediate end of the VM (phase III) the subjects could relax, the intrathoracic pressure returns close to the baseline level. Finally, in phase IV, an ABP overshoot is observed, due to the ejection of the blood into the constricted vasculature by the hearth following the end of the impediment of the venous return due to the VM. If the early gate rBFIs well represents the ABP pattern, we cannot affirm the same for the late gate, in particular for phase IV. In our results, for all the phases described above, late gate rBFI tends to show smaller changes as compared to early gate which might be the evidence of cerebral autoregulation influence while extracerebral (or early gate) rBFI is much more affected by the changes of the mean arterial pressure during VM exercise^37^. Pulsatility parameters also tend to demonstrate smaller changes from the baseline values during VM for the later time gate. A similar work on TD-DCS^46^ where they used another breathing exercise—breath hold—also reports a higher changes in the early gate rBFI compared to the late gate. Of course, this is just a preliminary measurement on a few subjects, the aim of which is to underline the possibility of enhancing the differences in the contribution between extra- and intra-cerebral blood flow signals, rather than to give a physiological conclusion.

In-vivo measurements are always limited by the maximum permissible exposure which limits the amount of power that can be used on the source side (wavelength dependent). Therefore, increasing the detection efficiency, parallelization and advancing data processing and analysis methods are the main ways of improving TD-DCS performance for in-vivo applications. In this regard, it is worth nothing that the field of SNSPDs is rapidly evolving, with recent development of devices with an extremely large number of pixels^48^, gating capability^43^, and extreme performances^25–27^. This makes them not only an already optimal solution for TD-DCS in laboratory settings for exploitative measurement campaigns, but also a dynamic field of research with potential to develop soon breakthrough technologies capable of revolutionizing the clinical environment^49^. However, SNSPDs are currently difficult to utilize in a clinical setting because of their bulkiness, power consumption, noise due to the cryostat/vacuum pump operation, and high cost as compared with SPADs. SPADs are extremely compact devices that permit the fabrication of wearable systems, but SNSPDs systems are progressively becoming more compact and less noisy, thus making feasible the arrangement of portable systems, suitable for the clinical environment^19^. As this type of works become commonplace, we believe that SNSPDs can be developed as more practical units since there are no physical or technological limitations. On the other side, the research on SPAD devices is advancing on efficiency, noise, afterpulsing, and response shape, with the potential to improve their performance in TD-DCS. Concerning the application, TD-DCS is an emerging field, with, to date, just few papers published. It is, therefore, largely unexplored in terms of simulations, measurement techniques, modeling, data analysis, and in-vivo tests. This combination of novelty and rapid advancements creates a dynamic and promising scenario in which it results difficult to draw robust and long lasting conclusions on best possible solutions for TD-DCS.

To conclude, in this work we have reported results of the measurements with a SNSPD-based TD-DCS system at 785 *nm* performed on Intralipid phantom and on adult human forehead. We have demonstrated the advantages of this system in comparison with TD-DCS measurements performed with a “classical” SPAD detector. We have also reported the capability of this system to extract gated pulsatile cerebral blood flow together with the optical parameters of the measured tissue. We have observed a difference in the hemodynamic response to *Valsalva maneuver* exercise between early and late time gates which might be the evidence of the increased brain sensitivity in case of the later time gate.

## Supplementary Information

Supplementary Information.

## Data Availability

The datasets used and analysed during the current study are available from the corresponding author on reasonable request.
