# Supplementary material for: Fast time-domain diffuse correlation spectroscopy with superconducting nanowire single-photon detector: system validation and in vivo results
Source: Sci Rep. 2023 Jul 24;13:11982. doi: 10.1038/s41598-023-39281-5 (PMC10366131; doi:10.1038/s41598-023-39281-5)
Supplement: Supplementary file 1 — Supplementary Information. [file 41598_2023_39281_MOESM1_ESM.pdf]

Supplementary materials

Title:

Fast time-domain diffuse correlation spectroscopy with superconducting nanowire single-photon detector: system validation and *in vivo* results

Authors:

Veronika Parfentyeva[1,+], Lorenzo Colombo[2,+], Pranav Lanka[2], Marco Pagliazzi[1], Annalisa Brodu[3], Niels Noordzij[3], Mirco Kolarczik[4], Alberto Dalla Mora[2], Rebecca Re[2,5], Davide Contini[2], Alessandro Torricelli[2,5], Turgut Durduran[1,6], and Antonio Pifferi[2,5]

Affiliations:

[1]Institut de Ciències Fotòniques, The Barcelona Institute of Science and Technology, 08860 Castelldefels, Barcelona, Spain

[2]Politecnico di Milano, Dipartimento di Fisica, Milano, 20133, Italy

[3]Single Quantum BV, Delft, 2629JD, The Netherlands

[4]Swabian Instruments GmbH, Stuttgart, 70435, Germany

[5]Consiglio Nazionale delle Ricerche, Istituto di Fotonica e Nanotecnologie, Milano, 20133, Italy

[6]Instituciò Catalana de Recerca i Estudis Avanc ̧ats (ICREA), Barcelona, 08015, Spain

+these authors contributed equally to this wo

Corresponding author:

alberto.dallamora@polimi.it

Figure S1 and Table S1 show results of *in vivo* measurements for the remaining subjects during Resting state period. Subject 3 demonstrated lower count rate in case of the late time gate comparing to other subjects which results in lower SNR and noisier BFI signal for that gate. These findings suggest that further optimisation of gating strategy implying individual subject approach is needed for in-vivo TD-DCS measurements.

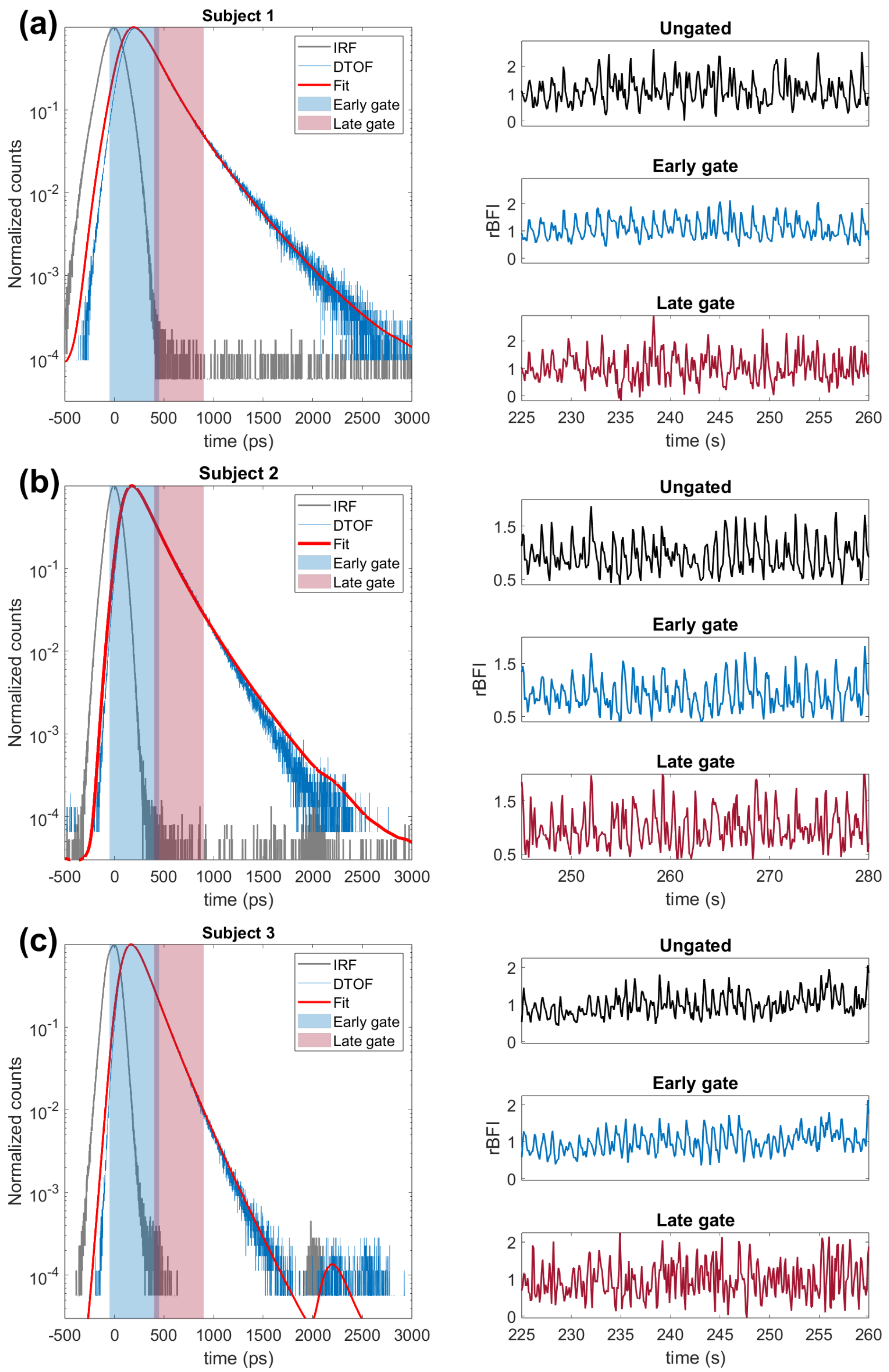

Figure S1. DTOF (blue), IRF (gray) and fitted DTOF curve (red) with shaded early and late time gates and rBFI for different time gates for a) Subject 1, b) Subject 2 and c) Subject 3 during Resting state.

Table S1. Count rate (in kcps) of in-vivo measurements of all subjects for different time gates.

Gate	S1	S2	S3	S4
Early gate	300	380	400	600
Late gate	80	80	60	100
Ungated	380	460	460	700
